# Characterization and monitoring of changes during lactation in the profile of multiple bioactive compounds of milk from grazing mares

**DOI:** 10.1002/jsfa.13966

**Published:** 2024-10-18

**Authors:** Ana Blanco‐Doval, Luis Javier R Barron, María Ángeles Bustamante, Noelia Aldai

**Affiliations:** ^1^ Lactiker Research Group, Department of Pharmacy and Food Sciences University of the Basque Country (UPV/EHU) Vitoria‐Gasteiz Spain

**Keywords:** hydrosoluble vitamin, liposoluble vitamin, pasture, polyphenol, antioxidant activity

## Abstract

**BACKGROUND:**

Mare milk has often been considered a food product with potential functional properties. However, the bioactive compound composition of mare milk, including vitamins and other minor bioactive compounds, as well as factors affecting this composition have scarcely been studied. Therefore, the present study aimed to characterize the changes during lactation in the content of water‐ and fat‐soluble vitamins and total polyphenols, and the total antioxidant capacity of mare milk from semi‐extensive farms. A total of 310 individual milk samples from 18 mares belonging to three commercial farms and 12 lactation times were analyzed. Ascorbic acid (vitamin C), thiamine (vitamin B_1_), riboflavin (vitamin B_2_), nicotinic acid and niacinamide (vitamins B_3_), pantothenic acid (vitamin B_5_), pyridoxal and pyridoxine (vitamins B_6_), folic acid (vitamin B_9_), cyanocobalamin (vitamin B_12_), tocopherols and tocotrienols (vitamin E) and retinol and retinyl esters (vitamin A) were quantified using liquid chromatography. Total polyphenols and antioxidant capacity assays were analyzed using spectrophotometry.

**RESULTS:**

The concentration of most bioactive compounds tended to decline as lactation progressed, with the exception of polyphenols and the total antioxidant capacity that oscillated during lactation. On the other hand, the effect of the different semi‐extensive management of the farms was only significant for vitamin B_3_ content.

**CONCLUSION:**

To the best of our knowledge, the present study provides the most in‐depth description of the vitamin profile of mare milk as well as new insights into polyphenol content and antioxidant capacity of mare milk. © 2024 The Author(s). *Journal of the Science of Food and Agriculture* published by John Wiley & Sons Ltd on behalf of Society of Chemical Industry.

## INTRODUCTION

Mare milk is a traditional dairy product in Central Asia and Eastern Europe, but its consumption has expanded to some other European countries during the last century.^1^, ^2^ Mare milk is highly valued for its composition similar to human milk, low allergenicity, lower casein to whey protein ratio^2^, ^3^ and its abundance in polyunsaturated fatty acids.^4^ Mare milk has traditionally been used for the prevention and treatment of several pathologies, the reason why its potential as a functional food product has been widely assumed.^1^, ^3^ Following such historical uses and assumptions, recent studies have moved towards studying mare milk functional properties in terms of fatigue,^5^ cancer,^6^ diabetes^7^ and wound healing,^8^ among others. However, the bioactive compound composition of mare milk has been substantially understudied compared to other milks, such as cow, sheep or goat milk. For instance, minor bioactive components including vitamins have been barely investigated, although some studies that address mare milk as a high‐value food product sometimes refer to its high vitamin content.^1^

Among the few available studies that analyzed this chemical fraction of the product, many of them are outdated but have been cited time after time due to the lack of recent research, as also discussed by Navrátilová *et al*.^9^ Most studies on mare milk water‐soluble vitamins used analytical methodologies such as microbiological, colorimetric or titration methods that only allowed an indirect approximation to the content.^10^, ^11^, ^12^, ^13^, ^14^ Small sample sizes and few influencing factors have often been considered,^11^, ^12^, ^15^, ^16^ and only a few studies accounted for changes during the lactation period,^9^, ^13^ using up to six sampling times and analyzing few vitamins.

Regarding fat‐soluble vitamins, the literature is also limited and studies have usually focused on a single isomer of vitamins A and E,^9^, ^13^, ^17^, ^18^, ^19^ providing an incomplete composition of mare milk, while changes in the content of these vitamins during lactation have been poorly studied.^9^ In addition, and to the best of our knowledge, how grazing affects the content of mare milk vitamins has not been investigated. In fact, pasture is overall rich in *α*‐tocopherol and *β*‐carotene, and previous research has shown an enhanced accumulation of these fat‐soluble compounds, as well as retinol, in milk from cows fed high pasture proportions.^20^ Therefore, exploring this relation in mare milk would be of great interest.

Polyphenols are plant secondary metabolites that exert antioxidant activity and contribute to the functional value of foods.^21^ Even though fruits and vegetables are the main dietary source of polyphenols,^21^, ^22^ these are also present in milk and dairy products,^23^ but to date their presence in mare milk has not been reported, while knowledge regarding the total antioxidant capacity of mare milk is also scarce. Characterization of these two parameters in mare milk is necessary for a better understanding of its composition and bioactivity, which in turn is essential to establish a basis for future research that evaluates mare milk as a functional food.

In the study presented here, a thorough characterization of bioactive compounds in mare milk was performed. This included a broad vitamin profiling, accounting for water‐ and fat‐soluble vitamins, and total polyphenol quantification, as well as the determination of total antioxidant capacity. Moreover, the effects of lactation (with 12 sampling times) and different feeding management systems (with different duration of the grazing period) were investigated in commercial equine farms.

## MATERIALS AND METHODS

### Experimental design and milk sampling

Individual milk samples were collected from 18 mares belonging to three different commercial farms (*n* = 6 mares per farm) located in Araba (northern Spain). All mares were from the Basque Mountain Horse breed. Mares were milked during the complete lactation period (6 months), once a week from early May to late July, and once every 14 days from early August to mid‐October. The semi‐extensive management system used for animal rearing differed among farms. Although the term ‘management system’ encompasses a number of different production factors such as the type of milking method, the milking hours, geographical location, etc., animal diet was considered as one of the most relevant factors that could potentially affect milk composition. In this sense, farm I used a pasture‐based system only during May (pasture composed of vetch, ryegrass and oat), and fed the animals with a mixture of alfalfa, silage, hay, fruits and potatoes (daily ration of 15–20 kg per mare) from June onwards. In contrast, farms II and III maintained a grazing management during the complete lactation period (pasture composed of alfalfa, clover, ryegrass, *Festuca* spp., orchard grass and dandelion), supplementing with hay (daily ration of 4–5 kg per mare) or grass and alfalfa silage (daily ration of 9–10 kg per mare), respectively, only after July in order to deal with low grass availability.

Before milking, foals were physically separated from their mothers for 2–3 h in order to prevent suckling and maximize milk accumulation in the udder. Then, mares were moved to a milking parlor and milked as completely as possible using either machine milking (farm I) or manual milking (farms II and III). During the milking process, the foals were kept in an adjoining area, so mares could feel and hear the foals close while preventing physical contact. A total of 310 individual milk samples were obtained for analysis of bioactive compounds and antioxidant capacity. After milking, the samples were immediately refrigerated (4 °C), transported to the laboratory, subsampled and preserved at −80 °C until analysis. Details of animal and sampling conditions are described in Blanco‐Doval *et al*.^24^ The commercial farms used standard milking procedures, and therefore institutional animal use approval was not required.

### Chemicals and reagents

#### Water‐soluble vitamins

Acetic acid glacial (Panreac, Barcelona, Spain), 1‐octanesulfonic acid sodium salt (ACROS Organics, Thermo Fisher Scientific, Waltham, MA, USA), triethylamine (Thermo Fisher Scientific), methanol for chromatographic analysis (Romil, Teknokroma, Barcelona, Spain) and potassium phosphate monobasic (Scharlab, Barcelona, Spain) were used for analysis. Ultrapure water was obtained from a Milli‐Q system (Merck Millipore, Darmstadt, Germany). Other chemicals were purchased from Merck (Darmstadt, Germany).

Precipitation solution was prepared with 9.1 g of zinc acetate dihydrate, 5.46 g of tungstophosphoric acid hydrate and 5.8 mL of acetic acid glacial in 100 mL of ultrapure water. The precipitation solution was prepared fresh every week. The aqueous mobile phase buffer was daily prepared and contained 6.8 g of potassium phosphate monobasic, 1.1 g of 1‐octanesulfonic acid sodium salt and 5 mL of triethylamine in 1 L of ultrapure water. Orthophosphoric acid was used to adjust the pH to 2.95.

High‐purity (>90%) commercial standards of calcium d‐pantothenate, cyanocobalamin, folic acid, nicotinamide, pyridoxine hydrochloride and riboflavin were purchased from Supelco (Merck), and l‐ascorbic acid, nicotinic acid, pyridoxal hydrochloride and thiamine hydrochloride from Sigma‐Aldrich (Merck).

#### Fat‐soluble vitamins

The reagents used for fat‐soluble vitamin analysis were isopropanol, *n*‐hexane and 1,4‐dioxane (Merck). High‐purity (>90%) commercial standards of *α*‐, *β*‐, *γ*‐ and *δ*‐tocopherol, *α*‐tocopherol acetate, *α*‐, *β*‐, *γ*‐ and *δ*‐tocotrienol, retinol, retinyl acetate, retinyl propionate and retinyl palmitate were purchased from Sigma‐Aldrich (Merck).

#### Total polyphenols

Acetonitrile was purchased from Romil (Teknokroma). Carrez I and II solutions were prepared according to manufacturer instructions. Carrez I solution was prepared with potassium hexacyanoferrate(II) trihydrate in distilled water (0.15 g mL^−1^). Carrez II solution was prepared with zinc sulfate heptahydrate in distilled water (0.30 g mL^−1^). All the chemicals used for Carrez I and II solutions were purchased from Merck.

#### Antioxidant capacity

A phosphate buffer solution (PBS; pH 7.4, 5 mmol L^−1^) was prepared with 4 g of sodium chloride, 100 mg of potassium chloride, 720 mg of disodium phosphate and 122.5 mg of potassium phosphate monobasic in 1 L of ultrapure water. The pH was adjusted with a 1 mol L^−1^ sodium hydroxide or hydrochloric acid solution. The 2′‐azinobis(3‐ethylbenzothiazoline‐6‐sulfonic acid) (ABTS) stock solution consisted of a solution of 7 mmol L^−1^ ABTS and 2.45 mmol L^−1^ potassium persulfate (final concentration) in ultrapure water. The solution was oxidized in agitation, dark conditions and room temperature for at least 12–16 h. ABTS^•+^ working solution was daily prepared by diluting the ABTS^•+^ stock solution in PBS until an absorbance of 0.70 ± 0.02 at 734 nm. All the chemicals used for ABTS^•+^ stock solution were purchased from Sigma‐Aldrich (Merck), except for potassium and sodium chloride (Panreac), potassium phosphate monobasic (Scharlab) and Trolox® reagent (ACROS Organics).

### Water‐soluble vitamin analysis

Water‐soluble vitamins were analyzed following the method by Zafra‐Gómez *et al*.^25^ with modifications. Briefly, mare milk was thawed in a water bath at 25 °C for 60 min. Around 5 ± 0.0001 g of liquid mare milk was mixed with 750 μL of the precipitation solution, and clarification was allowed for 15 min. Clarified samples were centrifuged for 5 min at 3500 × *g* and 20 °C using an ST 16R centrifuge (Sorvall, Thermo Fisher Scientific), and supernatants were filtered through 0.2 μm pore nylon filters (OlimPeak, Teknokroma). Filtered samples were transferred to 2 mL amber vials and kept at −80 °C until analysis. All the procedures were performed under dark conditions.

Water‐soluble vitamins were separated using a high‐performance liquid chromatograph (model 2695, Waters, Barcelona, Spain) coupled to fluorescence (model 2475 FLR, Waters) and diode array (model 2998 PDA, Waters) tandem detectors. For molecular separation, a C18 Spherisorb ODS‐2 column was used (25 cm long, 4.6 mm i.d., 3 μm particle size; Waters). Analytical conditions were as follows: 40 °C column temperature, 1 mL min^−1^ flow rate, 50 μL injection volume and gradient conditions of the mobile phase (phosphate buffer–methanol) as previously described by Zafra‐Gómez *et al*.^25^ The fluorescence detector was set at 290/410 nm (excitation/emission) wavelength for pyridoxine and pyridoxal, and 400/520 nm for riboflavin. The diode array detector was set at 245 nm for thiamine, 261 nm for nicotinic acid and niacinamide, 195 nm for pantothenic acid, 282 nm for folic and ascorbic acids and 370 nm for cyanocobalamin.

Water‐soluble vitamins were identified by comparing their retention times with those of high‐purity commercial standards. Quantification was done using external calibration curves created with four to six concentrations, depending on each vitamin. Ranges were as follows: 0.9–90 μg mL^−1^ for ascorbic acid; 0.005–0.5 μg mL^−1^ for cyanocobalamin; 0.05–5 μg mL^−1^ for folic acid; 0.02–2.5 μg mL^−1^ for nicotinic acid; 0.02–10 μg mL^−1^ for niacinamide; 0.2–6 μg mL^−1^ for pantothenic acid; 0.015–1.5 μg mL^−1^ for pyridoxal; 0.001–0.3 μg mL^−1^ for pyridoxine; 0.001–0.03 μg mL^−1^ for riboflavin; and 0.02–10 μg mL^−1^ for thiamine. Determination coefficient values (*R* ^2^) were higher than 0.999 except for riboflavin (0.957). Relative standard deviation (RSD) for six replicates of the standard solutions at a concentration range of 4.5 to 9.0 μg mL^−1^ for ascorbic acid and of 0.1 to 0.6 μg mL^−1^ for the rest of the vitamins was lower than 6.8%. Accuracy was evaluated in terms of recovery by analyzing three replicates of standard solutions, and the recovery values ranged between 80.4% and 104.0% for all water‐soluble vitamins except for pantothenic acid (73.4%) and cyanocobalamin (123.8%). The limit of quantification (LOQ) was determined experimentally for each vitamin. It was set as the lowest concentration at which the standard followed a linear correlation with the higher concentrations used for the calibration, and could be detected with RSD lower than 12% between triplicates. LOQs were 90 μg (100 g)^−1^ milk for ascorbic acid, 0.48 μg (100 g)^−1^ for cyanocobalamin, 5.0 μg (100 g)^−1^ for folic acid, 2.5 μg (100 g)^−1^ for nicotinic acid, 2.0 μg (100 g)^−1^ for niacinamide, 23 μg (100 g)^−1^ for pantothenic acid, 0.28 μg (100 g)^−1^ for pyridoxal, 0.13 μg (100 g)^−1^ for pyridoxine, 0.076 μg (100 g)^−1^ for riboflavin and 1.9 μg (100 g)^−1^ for thiamine. The concentrations of water‐soluble vitamins in mare milk samples were expressed as μg (100 g)^−1^ milk.

### Fat‐soluble vitamin analysis

Mare milk was thawed in a water bath at 25 °C for 60 min. Tocols (tocopherols and tocotrienols) and retinoids (retinol and retinyl esters) were simultaneously extracted from 2 ± 0.0001 g of liquid milk using two liquid–liquid extraction steps without saponification following the procedure described by Valdivielso *et al*.^26^ Tocols and retinoids were simultaneously analyzed using a high‐performance liquid chromatograph (model 1260, Agilent Technologies, Madrid, Spain) coupled to a fluorescence detector (1260 FLD Spectra, Agilent Technologies). Separation was done at 22 °C on a Luna Silica column (10 cm long, 3.0 mm i.d., 3 μm particle size; Phenomenex, Madrid, Spain) protected by a guard column and using a nonlinear gradient elution of 1,4‐dioxane/*n*‐hexane from 3/97 up to 25/75 (v/v) for 15 min as mobile phase. Then, the mobile phase was returned to initial conditions and the column was re‐equilibrated for 3 min. The flow rate was 1 mL min^−1^ and injection volume was 20 μL. The fluorescence detector operated at 298 nm for excitation wavelength, and 328 and 475 nm for emission wavelength, respectively, for tocols and retinoids. The compounds were identified by comparing the retention times of sample chromatographic peaks with those of high‐purity commercial standards. Quantification of tocols and retinoids in milk samples was done by external calibration using standard solutions in *n*‐hexane/isopropanol (99/1 v/v) analyzed in triplicate in four concentration levels from 0.01 to 0.5 μg mL^−1^. *R* ^2^ values of the linear calibrations were higher than 0.996. RSDs for five replicates of the standard solutions at the low and high concentrations were lower than 6.9%. In the same way, accuracy was evaluated in terms of recovery by analyzing six replicates of standard solutions at these concentrations, and the recovery values ranged between 88.1% and 110.9% for all tocols and retinoids studied. LOQs were determined by analyzing ten replicates of the lowest concentration of the standard solution that obtained an RSD value lower than 20%. This lowest concentration was established for all the tocols and retinoids in the milk samples as 0.750 μg (100 g)^−1^ milk. The concentrations of tocols and retinoids in mare milk samples were expressed as μg (100 g)^−1^ milk.

### Total polyphenol analysis

Total phenolic content of mare milk was measured using the Folin–Ciocalteu method adapted for milk by Vázquez *et al*.^27^ In brief, milk samples were thawed in a water bath at 37 °C for 45 min under dark conditions. Then, milk was homogenized through agitation, 8 ± 0.0001 g was weighed and 10 mL of methanol/distilled water (1/1, v/v), 500 μL of Carrez I solution, 500 μL of Carrez II solution and 5 mL of acetonitrile were added for clarification. The solution was adjusted to 25 mL using methanol/distilled water (1/1, v/v) and was left for 25 min in darkness until complete clot precipitation. Phases were separated by centrifugation for 15 min at 4700 × *g* and 20 °C using an ST 16R centrifuge (Sorvall, Thermo Fisher Scientific). The supernatant (polyphenol extract) was collected. In dim light and at room temperature, 60 μL of extract, standard solution or ultrapure water (blank), 250 μL of ultrapure water and 65 μL of Folin–Ciocalteu reagent (2 N) were mixed in 2 mL microcentrifuge tubes. Then, 625 μL of sodium carbonate solution (7% w/v) and 500 μL of ultrapure water were added. Solutions were allowed to react for 90 min in dark conditions and at room temperature. Absorbance was read at 750 nm with a Cary 50 Bio UV‐VIS spectrophotometer (Varian, Agilent Technologies).

Total polyphenol concentrations in mare milk were quantified using external calibration. A calibration curve was built with gallic acid standard solutions in methanol/water (1/1, v/v) at five concentration levels from 1.25 to 250 μg mL^−1^. Mean *R* ^2^ values of the linear calibrations were 0.9995. Blanks were done with ultrapure water instead of standard or extract (no differences were observed when using methanol/water (1/1, v/v) as blank; data not shown). RSD for five replicates of polyphenol extraction and analysis was 2.82%. The concentrations of polyphenols in mare milk samples were expressed as mg of gallic acid equivalents (GAE) per 100 g of milk.

### Determination of total antioxidant capacity

The total antioxidant capacity of mare milk was determined according to the procedure of Gila‐Díaz *et al*.,^28^ with minor modifications. Mare milk samples were thawed in a water bath at 25 °C for 60 min and diluted 1/20 (v/v) in PBS (75 μL of milk and 1.425 mL of PBS). For spectrophotometric analysis, 150 μL of diluted sample, standard or PBS (control) were mixed with 1350 μL of ABTS^•+^ working solution. For turbidity corrections, 150 μL of each sample was diluted in 1350 μL of PBS. Reaction was allowed for 10 min at 25 °C under dark conditions. Absorbance was read at 734 nm with a Cary 50 Bio UV‐VIS spectrophotometer (Varian, Agilent Technologies). Absorbance of individual milk samples was corrected with the corresponding turbidity absorbance value.

To measure the contribution of caseins to whole mare milk antioxidant capacity, the whey fraction was isolated through isoelectric precipitation of caseins based on the method by Ochirkhuyag *et al*.,^29^ with some modifications. For this, 10 ± 0.0001 g of 20 randomly selected mare milk samples was weighed, and lactic acid (10%, v/v) was added until the pH was 4.2 (isoelectric point of mare milk caseins according to Egito *et al*.^30^). Then, samples were centrifuged at 3500 × *g* and 22 °C for 30 min in an ST 16 R centrifuge (Sorvall, Thermo Fisher Scientific). Supernatants were collected and casein pellets were washed with 10 mL of ultrapure water at pH 4.2 (pH adjusted with 1 mol L^−1^ hydrochloric acid). The supernatant was pooled with the first extracted whey fraction, and it was diluted 1/10 (v/v) in PBS. Analysis was performed as for whole milk.

Total antioxidant capacity was estimated as Trolox equivalents (mmol L^−1^) and as radical scavenging activity (percentage of ABTS^•+^ radical cation inhibition). Quantification (Trolox equivalents) was done with calibration curves constructed using five calibration levels of standard Trolox solutions at different concentrations from 0.01 to 0.25 mmol L^−1^ in PBS. Mean *R* ^2^ values of the linear calibrations were 0.994. Percentage of inhibition was estimated using the following equation:

$$\text{Inhibition}\;\left(\%\right)=\left[\frac{A_{\text{control}}-A_{\text{sample}}}{A_{\text{control}}}\right]\times 100$$

where *A*_control_ is the absorbance of PBS (with no sample or standard added) and *A*_sample_ is the absorbance of the sample corrected with the absorbance of turbidity. Total antioxidant capacity of mare milk samples was expressed as Trolox equivalents (mmol L^−1^) and as percentage of inhibition. RSD was 0.53% for five replicates of whole milk analysis and 2.84% for five replicates of whey fraction separation and subsequent analysis.

### Statistical analysis

Statistical analysis was performed using IBM‐SPSS Statistics software version 28.0 (IBM, Endicott, NY, USA). Briefly, all data were log transformed, and values higher than three times the interquartile range and values below LOQ were identified as outliers and excluded from the dataset (excluded values were treated as missing values). The data were pooled in ranges of 14 days maintaining animal individuality, resulting in 12 lactation times (earliest lactation time at weeks 3 and 4, and latest at weeks 25 and 26). Lactation time was estimated based on parturition dates of individual mares. Data from the analysis of ascorbic acid, pantothenic acid, niacinamide, pyridoxal, folic acid, pyridoxine, cyanocobalamin, riboflavin, *α*‐tocopherol, retinyl palmitate, total polyphenols and antioxidant capacity were subjected to a linear mixed model of analysis of variance. The model included individual animal as subject, farm as fixed factor and lactation time as repeated measure factor, and the interaction effect between lactation stage and farm was also included. Parameters were estimated using the restricted maximum likelihood method, and the repeated measures covariance structure was built according to the compound symmetry matrix using the Akaike information criterion. In addition, the same data (except for folic acid) were subjected to multivariate analysis using a stepwise discriminant analysis in order to classify samples according to farm or lactation stage. In this case, lactation stage was defined as follows: early lactation, weeks 3 to 10; mid lactation, weeks 11 to 18; late lactation, weeks 19 to 26. Significance level was declared at *P* ≤ 0.05. Results were expressed to three significant figures.

## RESULTS

### Content of bioactive compounds and antioxidant capacity of mare milk

Thiamine, retinyl acetate, retinyl propionate, retinol, *α*‐tocopherol acetate, *β*‐, *γ*‐ and *δ*‐tocopherol and *α*‐, *β*‐, *γ*‐ and *δ*‐tocotrienol were not found in mare milk samples or were found below the LOQ. From the 310 milk samples analyzed, only 84 presented nicotinic acid contents above the LOQ. These 84 samples were randomly distributed during lactation, among farms and among individual animals. Therefore, the vitamin profile of mare milk was limited to ascorbic acid, pantothenic acid, niacinamide, pyridoxal and pyridoxine, folic acid, cyanocobalamin and riboflavin among water‐soluble vitamins, and *α*‐tocopherol and retinyl palmitate among fat‐soluble vitamins.

The content of bioactive compounds (water‐ and fat‐soluble vitamins and total polyphenols) and the antioxidant capacity of mare milk are summarized in Table 1. The content of water‐soluble vitamins was considerably higher than that of fat‐soluble vitamins. Among water‐soluble vitamins, the major one was ascorbic acid, showing an average concentration of 1,184 ± 941 μg (100 g)^−1^ milk. This was followed by a considerable amount of pantothenic acid (366 ± 348 μg (100 g)^−1^ milk), whereas other water‐soluble vitamins were found at much lower concentrations. Average contents of minor water‐soluble vitamins per 100 g of milk were 19.8 ± 15.6 μg for niacinamide, 14.2 ± 5.8 μg for pyridoxal, 7.57 ± 1.78 μg for folic acid, 2.25 ± 1.74 μg for pyridoxine, 1.51 ± 0.24 μg for cyanocobalamin and 1.16 ± 0.87 μg for riboflavin.

**Table 1:** Average concentration and range (minimum–maximum) of bioactive compounds in mare milk samples expressed as μg (100 g)^−1^ milk (vitamins), mg GAE (100 g)^−1^ milk (polyphenols) or mmol L^−1^ Trolox equivalents (total antioxidant capacity), and statistical significance (*P* value) of farm, lactation and interaction effects

Bioactive compound	Farm							*P*		
	I	*n*	II	*n*	III	*n*	SEM	Farm	Lactation	Farm × lactation
*Water‐soluble vitamins*										
Ascorbic acid	127 × 10^1^ (261–498 × 10^1^)	67	126 × 10^1^ (303–377 × 10^1^)	112	104·10^1^ (147–620 × 10^1^)	95	57	0.092	0.252	0.719
Pantothenic acid	375 (26.7–136 × 10^1^)	70	302 (38.4–168 × 10^1^)	108	431 (33.2–284 × 10^1^)	95	21	0.056	<0.001	0.713
Niacinamide	13.2^b^ (2.41–37.5)	60	24.5^a^ (2.27–105)	104	18.8^a^ (2.79–67.0)	90	1.0	0.012	<0.001	0.477
Pyridoxal	13.6 (3.22–29.8)	69	15.1 (4.81–28.8)	112	13.7 (4.31–24.1)	96	0.3	0.098	<0.001	0.031
Folic acid	8.04 (5.01–12.7)	56	7.66 (5.00–13.0)	75	7.04 (5.10–12.8)	60	0.13	0.061	<0.001	0.700
Pyridoxine	2.61 (0.593–18.8)	70	2.19 (0.265–8.65)	112	2.06 (0.285–10.0)	96	0.10	0.475	0.098	0.161
Cyanocobalamin	1.51 (1.02–2.04)	70	1.53 (1.03–2.30)	112	1.49 (0.932–2.17)	96	0.01	0.479	0.010	0.106
Riboflavin	0.786 (0.0902–3.16)	57	1.35 (0.0904–4.96)	108	1.16 (0.0760–4.69)	94	0.054	0.273	0.004	0.112
*Fat‐soluble vitamins*										
*α*‐Tocopherol	10.4 (2.87–59.7)	69	10.8 (1.92–37.3)	112	10.2 (2.68–37.5)	94	0.4	0.542	<0.001	0.076
Retinyl palmitate	3.64 (0.678–10.7)	69	3.67 (0.619–12.0)	112	3.66 (0.655–20.5)	96	0.14	0.896	<0.001	0.927
Total polyphenols	7.61 (3.94–11.2)	66	7.08 (3.88–11.2)	112	6.90 (4.09–9.68)	96	0.09	0.519	<0.001	0.159
Total antioxidant capacity	3.95 (2.64–4.47)	66	3.84 (2.55–4.52)	112	3.87 (2.81–4.88)	96	0.03	0.889	<0.001	0.179

Total numbers of milk samples collected in commercial farms were 81, 125 and 104 for farms I, II and III, respectively. ^a,b^ Different superscripts refer to significant differences (*P* ≤ 0.05) among farms. *n*, number of milk samples from each farm in which the bioactive compounds and antioxidant capacity values were found to be above the limit of quantification.

GAE, gallic acid equivalents; SEM, standard error of the mean.

Among all the vitamin E isoforms studied, only one (*α*‐tocopherol) was present in mare milk samples above the LOQ. This was, in fact, the main fat‐soluble vitamin identified in the present study (10.5 ± 6.6 μg (100 g)^−1^ milk). Regarding vitamin A, retinol was only found as retinyl palmitate, showing an average concentration of 3.66 ± 2.34 μg (100 g)^−1^ milk. The non‐esterified molecule (retinol) was not quantifiable in most mare milk samples.

The average content of total polyphenols in mare milk samples was 7.15 ± 1.42 mg GAE (100 g)^−1^ milk, and the total antioxidant capacity was on average 3.88 ± 0.41 mmol L^−1^ of Trolox equivalents, resulting in an average Trolox inhibition of 82.1 ± 7.3%. After casein precipitation at pH 4.2 (whey fraction), the capacity of milk to inhibit Trolox almost halved compared to values observed in whole milk. Thus, caseins contributed to 49.9 ± 8.0% of the total antioxidant capacity of mare milk.

### Effect of management system and lactation stage

**Figure 1:**
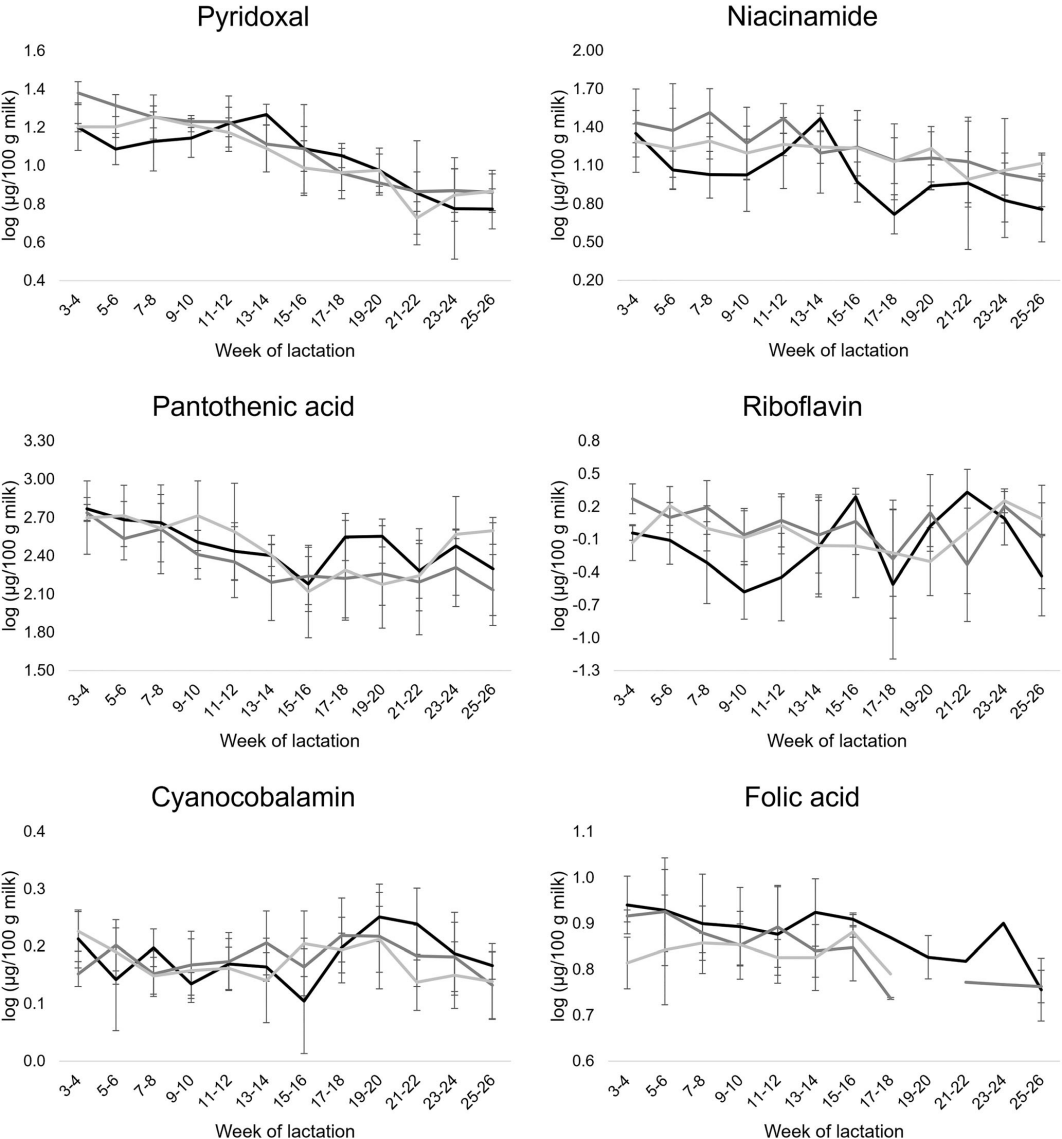
Changes during lactation in the content of water‐soluble vitamins in mare milk samples belonging to three commercial farms: 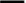, farm I; 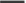, farm II; 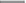, farm III.

**Figure 2:**
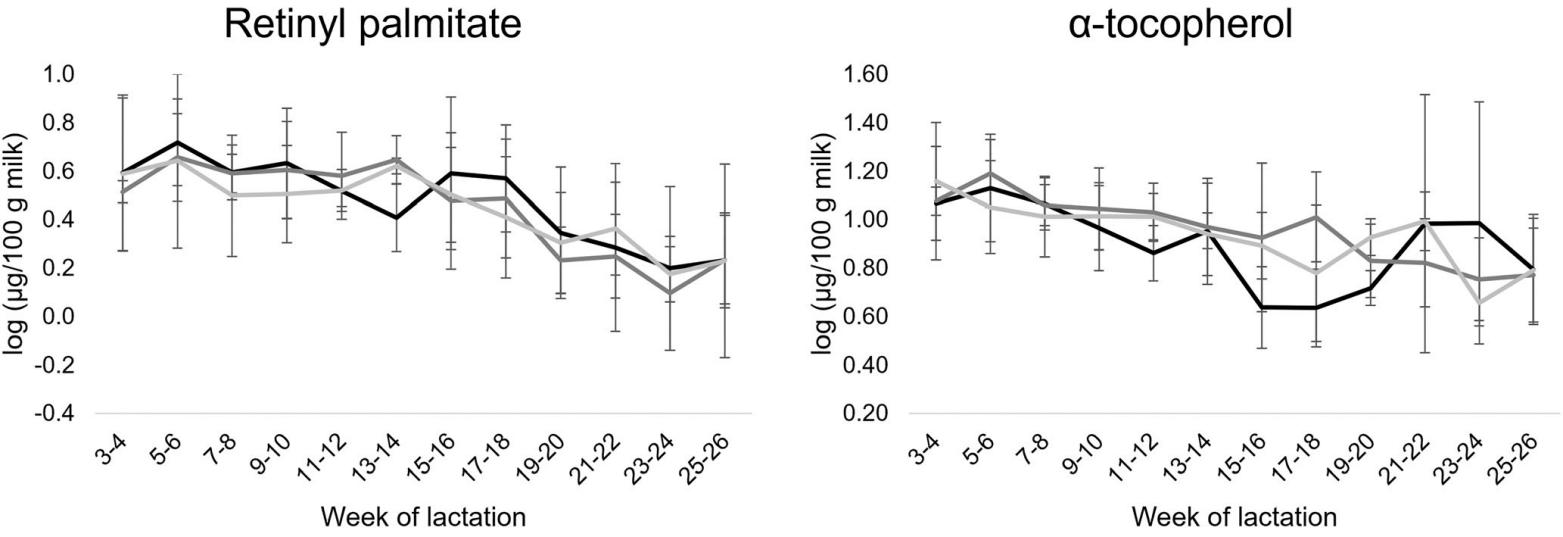
Changes during lactation in the content of fat‐soluble vitamins in mare milk samples belonging to three commercial farms: 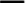, farm I; 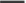, farm II; 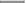, farm III.

**Figure 3:**
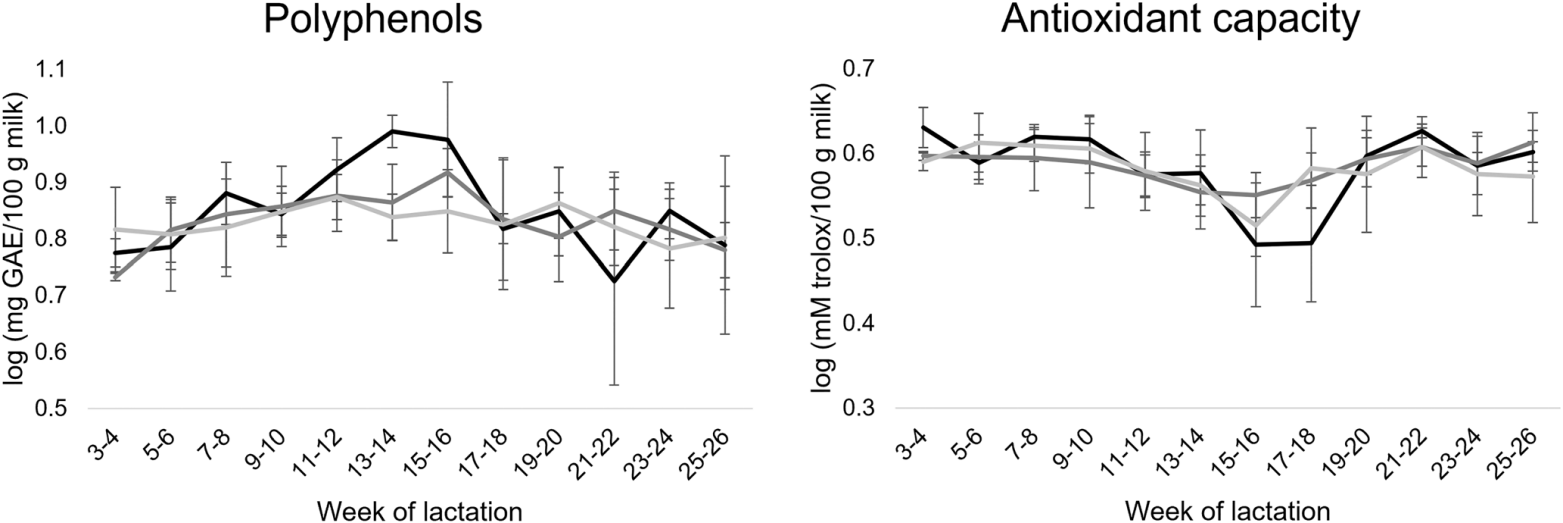
Changes during lactation in the polyphenol content and total antioxidant capacity in mare milk samples belonging to three commercial farms: 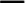, farm I; 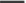, farm II; 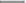, farm III.

The effect of lactation stage and management system (farm) is presented in Table 1. Farm effect was only significant (*P* ≤ 0.05) for niacinamide content, which was higher in milk from farms with a long grazing period (farms II and III) compared to a short grazing period (farm I). The lack of significant effect among milks from different farms in most compounds analyzed is more than likely due to the large variability observed among milk samples within each farm (Table 1), evidencing a strong individual animal effect on mare milk bioactive compounds. On the other hand, lactation stage significantly (*P* ≤ 0.05) affected the content of most water‐ and fat‐soluble vitamins analyzed, except for ascorbic acid and pyridoxine. The effect of lactation stage was also significant (*P* ≤ 0.05) for total polyphenols and the total antioxidant capacity of milk (Table 1). Figures 1, 2, 3 show the changes during lactation in the content of individual water‐ and fat‐soluble vitamins and total polyphenols, as well as in the values of the total antioxidant capacity of mare milk samples. In terms of vitamins, the content of pyridoxal, niacinamide, pantothenic acid, retinyl palmitate and *α*‐tocopherol decreased from early (weeks 3 and 4) to late (weeks 25 and 26) lactation. For niacinamide and retinyl palmitate, the decrease was more evident after weeks 13–16 of lactation, whereas pantothenic acid contents mainly decreased from early lactation to weeks 15 and 16 with moderate changes afterwards (Figs 1 and 2). On the other hand, riboflavin and cyanocobalamin contents remained quite constant but with fluctuations during lactation. Folic acid contents remained stable from early lactation until weeks 13 and 14. Then, decreased to values below the LOQ in most milk samples independently of farm and lactation time (Fig. 1). Regarding total polyphenols, these peaked at mid lactation (weeks 11–18), but similar values were found at early and late lactation stages (Fig. 3). Interestingly, the opposite pattern was observed in the total antioxidant capacity of mare milk, which was lowest at mid lactation (weeks 15–18). The interaction effect between farm and lactation stage was only significant for pyridoxal contents (Table 1). In this respect, milk from mares in a short grazing period (farm I) followed a different pattern during lactation compared to milk from mares with a long grazing period (farms II and III) (Fig. 1).

**Figure 4:**
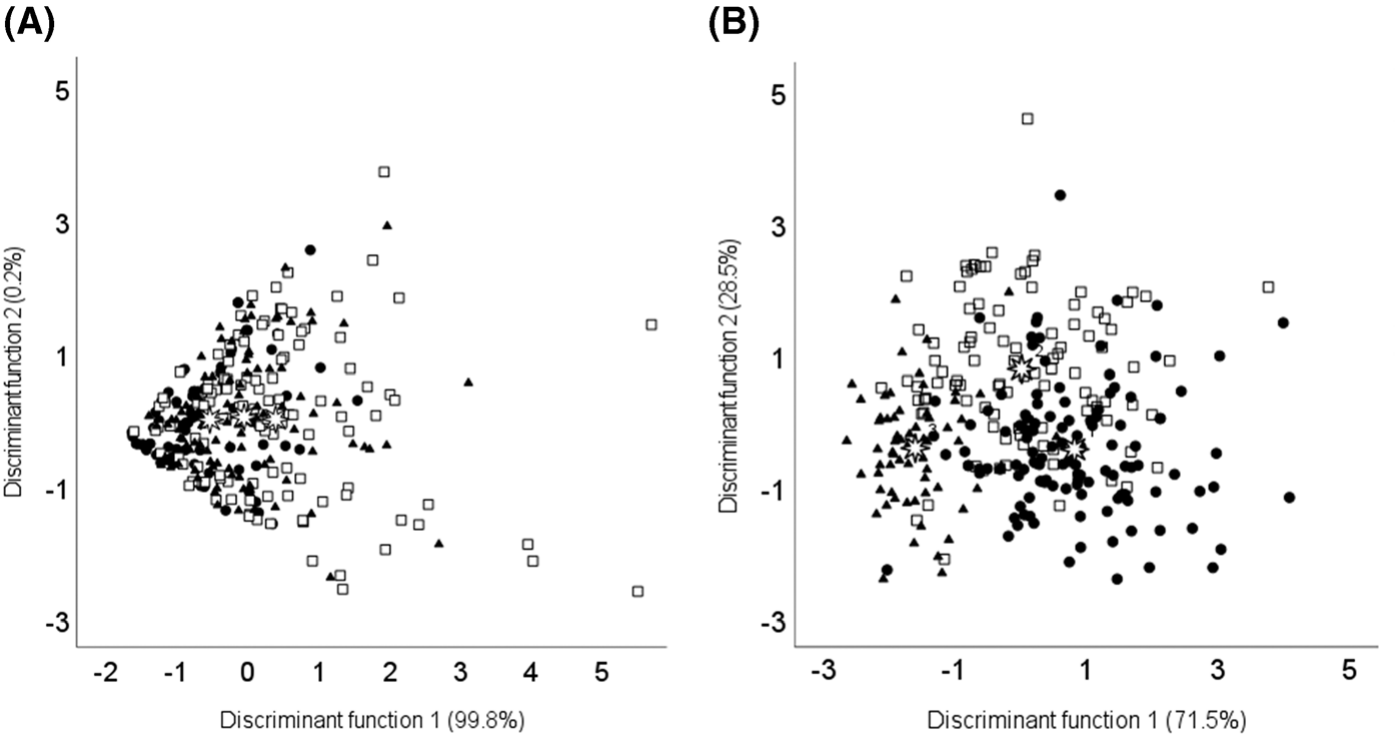
Graphical representation of the two first canonical discriminant functions for stepwise discriminant analysis of mare milk bioactive compounds and antioxidant capacity based on (A) management system (●, farm I; □, farm II; ▲, farm III) and (B) lactation stage (●, early lactation; □, mid lactation; ▲, late lactation). 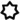, group centroid.

The multivariate analysis confirmed the effect of lactation stage on the content of mare milk bioactive compounds, whereas milk samples were not effectively discriminated according to farm. Canonical distributions of mare milk samples in the first two canonical functions obtained according to management system (farms I, II and III) or lactation stage (early, mid and late lactation) are depicted in Fig. 4.

Regarding the effect of lactation stage, 79.5% and 84.1% of the samples from early and late lactation, respectively, were correctly classified into their corresponding groups. However, milk samples from mid lactation were mixed with the other two groups. After cross‐validation, 74.0% of classification was obtained. Function 1 axis (Fig. 4) was the function that discriminated mainly between samples from early and late lactation, and was mainly correlated with pyridoxal, retinyl palmitate and *α*‐tocopherol contents in mare milk samples. This is in agreement with a clear decreasing trend in the content of these three vitamins during lactation (Figs 1 and 2). On the other hand, function 2 axis discriminated mid lactation from early and late lactation samples, and was mainly correlated with the total antioxidant capacity and total polyphenol content. These results supported the evolution trends depicted in Fig. 3, which showed maximum and minimum values at mid lactation respectively for total polyphenols and total antioxidant capacity.

## DISCUSSION

### Fat‐ and water‐soluble vitamins

In the present study, the content (in absolute basis) of water‐soluble vitamins in mare milk was considerably higher than that of fat‐soluble vitamins. Mare milk is very poor in fat compared to milk from ruminant species.^31^ Particularly, milk from the Basque Mountain Horse breed contains up to 3.06 g fat kg^−1^ milk (data not reported), limiting the accumulation of fat‐soluble vitamins.

According to the literature, vitamins A and E are present in greater concentrations than vitamins D and K.^31^ The present study corroborates that, in mare milk, vitamin E is more abundant than vitamin A as also evidenced by others.^9^, ^15^, ^16^ Csapó *et al*.^15^ reported higher values of fat‐soluble vitamins in mature milk than those found in the present study, which could be due to a higher total fat content in milk (12.5 g kg^−1^ milk) or milk being from early lactation stages (up to 6 weeks of lactation), since a decrease in the content of fat‐soluble vitamins during lactation was demonstrated in the present study. However, high variability regarding fat‐soluble vitamin content in mare milk has been observed in the literature,^9^, ^16^, ^32^, ^33^ which could be a consequence of different experimental and analytical conditions among studies.

Among the eight isomers that conform vitamin E (*α*‐, *β*‐, *γ*‐ and *δ*‐tocopherol and *α*‐, *β*‐, *γ*‐ and *δ*‐tocotrienol), mare milk in the present study mainly contained *α*‐tocopherol, whereas other isoforms were not detected. This was also observed by Marconi and Panfili.^16^ In the case of retinoids, only retinyl palmitate was present in mare milk, whereas free retinol or other esterified forms were almost negligible. According to Stowe,^34^ retinol in mare milk is mainly present as an ester. Vitamin A or retinol is a product of the hydrolysis of its precursor *β*‐carotene. Plants are a good source of *β*‐carotene,^35^ and horses, as well as cattle, are quite efficient at absorbing dietary carotenoids, unlike goat and sheep.^36^ When animals consume plants, an enzyme from the intestinal mucosa, named *β*‐carotene‐15,15′‐monooxygenase, converts the precursor molecule into all‐*trans*‐retinal (precursor of the active form retinol), and then retinol is esterified in the mammary gland.^35^, ^37^ Therefore, retinoids are mainly transferred into milk as esters.

In contrast to results by Navrátilová *et al*.^9^ that revealed a decreasing but not significant trend of fat‐soluble vitamins during mare lactation, we found a significant (*P* ≤ 0.05) decrease, which is in accordance with a significant reduction of the total fat content in mare milk.^38^ Stowe^34^ suggested that high vitamin A concentrations in mare colostrum could respond to the needs of the neonate. This might be extrapolated to the complete lactation period as a response to a decreasing reliance of a foal on milk as it consumes more and more feedstuff. On the other hand, different management systems did not result in different fat‐soluble vitamin contents in the present study, probably because all the three diets contained some kind of forages (fresh pasture or alfalfa) that are a good source of vitamins or vitamin precursors.^35^, ^39^ However, a high intra‐farm variability was observed, reflecting how individual animal strongly influences the abundance of fat‐soluble vitamins in mare milk.

Regarding water‐soluble vitamins, these are generally less abundant in mare milk than in ruminant milk.^31^ In ruminants, water‐soluble vitamins are synthesized in the rumen^40^ and, therefore, equids might have the synthesis‐assimilation of water‐soluble vitamins limited. Moreover, each vitamin has a particular metabolism^35^, ^40^ that leads to different transfer rates into milk, also affected by different responses to external factors (such as diet and lactation stage).

Overall, lower riboflavin contents were found in mare milk in the present study compared to previous reports.^9^, ^10^, ^11^, ^14^ Differences between studies could come from variability among analytical methods (some of the methods used for vitamin quantification were indirect and of low accuracy), or from milk characteristics. However, the very scarce literature on mare milk water‐soluble vitamins makes comparison difficult. This is the case for pantothenic acid, pyridoxine, folic acid and cobalamin, which have been reported only in one or two studies that used enzymatic, microbiologic or spectrophotometric assays (except for pyridoxine that was determined by liquid chromatography).^9^, ^11^, ^12^, ^14^ In the case of ascorbic acid, most reported values for mare milk are similar to or slightly higher than those reported in this study.^10^, ^14^, ^15^ Thiamine was not found in mare milk samples in the present study. Thiamine in ruminant milk mainly comes from ruminal synthesis (in the foregut)^35^ and absorption in the small intestine. However, in monogastrics thiamine is synthesized by the microbiota in the large intestine (hindgut fermentation),^41^ after the small intestine (the main absorption site). Therefore, lower absorption rates and transfer to milk are expected in monogastrics, as confirmed by the present study. Niacin or vitamin B_3_ is made up of two vitamers: nicotinic acid and nicotinamide (the amide form of nicotinic acid^41^). In mare milk, only few studies determined niacin. These studies analyzed nicotinic acid only, with no mention of nicotinamide, and used microbiological methods for quantification.^10^, ^11^ Both nicotinic acid and niacinamide are absorbed from diet, although niacinamide can also be produced by mucosal enzymes and nicotinic acid from deamination of niacinamide by gut microbiota.^42^ In this regard, the present study demonstrated that the main vitamin B_3_ form in mare milk was niacinamide, as also found in cow milk.^35^ Similarly, pyridoxal contents were on average more than five times higher than pyridoxine contents, which is in agreement with pyridoxal being the main vitamin B_6_ form in ruminant milk.^35^

The present study demonstrated a significant impact of lactation stage on most mare milk water‐soluble vitamins. The decreasing trend in vitamin concentrations in mare milk as lactation advances follows the pattern previously described for some macrominerals of the same milk samples.^24^ As confirmed by a multivariate statistical analysis, most evident changes were observed between early and late lactation stages and mainly due to variations in pyridoxal contents. There was a significant interaction between farm and lactation stage for pyridoxal, which in fact peaked at some point during mid lactation only in samples from mares in a short grazing period (farm I), whereas it continuously decreased in those from mares in a long grazing period (farms II and III). Therefore, a longer grazing period could modify the content of vitamin B_6_ in mare milk during lactation. Similarly, Navrátilová *et al*.^9^ observed that pyridoxine contents in milk from Warmblood mares without grazing peaked at mid lactation and decreased to minimum values after six months of lactation. However, only niacinamide was significantly affected by management of the mares, milk samples from mares in a long grazing period being richer in this vitamin. Niacin can be synthesized from l‐tryptophan, and therefore not only the niacin content but also the protein content and profile of the feeding administered to animals could affect niacin in milk.^41^

### Total polyphenols and antioxidant capacity

Some studies of ruminant milk observed an increase in the total phenolic content when animals were reared under pasture‐based systems^43^, ^44^ due to the high polyphenol content and diversity of pasture grasses and legumes.^45^ However, the management system used for mare milk production did not influence the content of phenolic compounds in milk in the present study. This was probably because the addition of forages, fruits and potatoes into the low grazing diet balanced the intake of dietary polyphenols among farms. For instance, fruits are a great source of phenolic compounds,^21^, ^22^ while changes in the phenolic content of milk during lactation can be attributed to seasonal variations of the phenolic profile of pastures or forage species.^45^

On the other hand, the total antioxidant capacity of mare milk samples (inhibition of 82%) was similar to that observed by Cosentino *et al*.^46^ in milk from Murgese mares (86–90%). Caseins are important contributors to milk antioxidant capacity.^47^ Specifically, the present study showed that after casein precipitation, the antioxidant capacity of milk halved. Therefore, the decrease in total antioxidant capacity from early to mid lactation could be linked to a decrease in the total protein content in mare milk.^48^ However, milk is a complex matrix, and a number of antioxidant compounds can also be present in the whey fraction of milk. Vitamins E and C are well‐known antioxidants,^49^, ^50^ but other compounds such as vitamin A,^50^ vitamin D_3_, coenzyme Q_10_, some whey proteins^47^ and polyphenols,^21^ among others, also contribute to the antioxidant activity. Dynamics of individual antioxidant compounds during lactation are probably responsible for changes in the antioxidant capacity of milk during lactation, particularly after mid lactation when it increases. In this case, polyphenols in mare milk are probably poor contributors to total antioxidant capacity, since they changed differently from antioxidant capacity during lactation. Among the studied parameters, total polyphenols and antioxidant capacity were the ones that mainly changed during mid lactation.

Regarding the effect of the management system, milk from the three commercial farms presented a similar total antioxidant capacity among milk samples from different farms. These results differ from those of studies in ruminant milk that observed an improved antioxidant capacity of milk when animals grazed.^43^, ^44^ In the present study, a shorter grazing period in farm I could have been compensated, in terms of antioxidant capacity, with the inclusion of ingredients rich in direct or indirect antioxidant compounds, for example, fruits and potatoes that are rich in vitamins A, E and C, minerals and phenolic compounds.^21^, ^22^, ^51^, ^52^

The present study addressed the characterization of mare milk vitamins and other bioactive compounds using a local horse breed. However, it is still not clear whether breed can affect mare milk vitamin composition. For instance, some authors found no differences in vitamin C content between milk from Thoroughbred and Polish Half Bred mares,^13^ whereas other studies did not consider different horse breeds for the study of milk vitamins. In this context, studies addressing the effect of breed on mare milk composition would be necessary to better understand mare milk quality in terms of bioactive compounds.

## CONCLUSION

To the best of our knowledge, the present study provides the most in‐depth characterization of mare milk bioactive compounds and antioxidant capacity to date, and elucidates changes during lactation and among different management systems. The content of some of the vitamins studied was clearly different between early and late lactation, whereas total polyphenols and antioxidant capacity of mare milk were different mainly during mid lactation. Conversely, the management system had a low impact on mare milk bioactive compounds. In addition, the present study demonstrates that the antioxidant capacity of mare milk is greatly influenced by milk caseins, whereas polyphenols are probably poor contributors.

This study updates and deepens the understanding of mare milk composition with a special focus on compounds that exhibit biological functions, and establishes a basis for future research on the value of mare milk as a functional food.

## CONFLICT OF INTEREST

None.

## Data Availability

The data that support the findings of this study are available from the corresponding author upon reasonable request.
